# Staining of activated ß_2_-integrins in combination with CD137 and CD154 for sensitive identification of functional antigen-specific CD4^+^ and CD8^+^ T cells

**DOI:** 10.3389/fimmu.2022.1107366

**Published:** 2023-01-19

**Authors:** Anna Schöllhorn, Ana Maia, Felix Kimmerle, Jan Born, Hans-Georg Rammensee, Stoyan Dimitrov, Cécile Gouttefangeas

**Affiliations:** ^1^ Department of Immunology, Institute for Cell Biology, University of Tübingen, Tübingen, Germany; ^2^ Institute of Medical Psychology and Behavioral Neurobiology, University of Tübingen, Tübingen, Germany; ^3^ German Center for Diabetes Research (DZD), Tübingen, Germany; ^4^ Institute for Diabetes Research and Metabolic Diseases of the Helmholtz Center Munich, University of Tübingen (IDM), Tübingen, Germany; ^5^ German Cancer Consortium (DKTK) and German Cancer Research Center (DKFZ) partner site Tübingen, Tübingen, Germany; ^6^ Cluster of Excellence iFIT (EXC2180) “Image-Guided and Functionally Instructed Tumor Therapies”, University of Tübingen, Tübingen, Germany

**Keywords:** integrin activation, cell surface staining, activation induced marker (AIM), antigen-specificity, CD4+ T cells, CD8+ T cells, CD154, CD137

## Abstract

Common flow cytometry-based methods used for functional assessment of antigen-specific T cells rely on *de novo* expression of intracellular cytokines or cell surface activation induced markers. They come with some limitations such as complex experimental setting, loss of cell viability and often high unspecific background which impairs assay sensitivity. We have previously shown that staining of activated ß_2_-integrins either with multimers of their ligand ICAM-1 or with a monoclonal antibody can serve as a functional marker detectable on T cells after minutes (CD8^+^) or few hours (CD4^+^) of activation. Here, we present a simple method for detection of activated ß_2_-integrins in combination with established cell surface activation induced markers. We observed that activated ß_2_-integrins were still detectable after 14 hours of stimulation, allowing their detection together with CD137 and CD154. Combinatorial gating of cells expressing activated ß_2_-integrins and CD137 or CD154 reduced background in unstimulated samples, increasing the signal-to-noise ratio and allowing improved assessment of low-frequency T cell responses. Extracellular staining of these markers highly correlated with production of intracellular cytokines IL-2, TNF or IFNγ in CD4^+^ and CD8^+^ T cells. As an exemplary application, SARS-CoV-2 spike-specific T cell responses were assessed in individuals after COVID-19 vaccination. This method should be useful for epitope discovery projects and for the simultaneous monitoring of low-frequency antigen-specific CD4^+^ and CD8^+^ T cell responses in various physiological situations.

## 1 Introduction

CD4^+^ and CD8^+^ T cells play an essential role in immunity against infectious diseases and cancer. For both basic and translational research (e.g. epitope mapping or monitoring of clinical specimens), testing of antigen-specific T cells should be sensitive enough to detect and quantify low-frequency antigen-specific T cells, robust and reproducible, as well as applicable to high numbers of tests and limited amount of material. Flow cytometry is commonly used to assess antigen-specific T cells, as it presents the unique advantage to deliver multiparametric, phenotypic and functional information at a single cell level while allowing to routinely analyze millions of cells in a short time (1, 2). A common method for assessment of T cell functionality is intracellular cytokine staining (ICS) for detection of e.g. IFNγ, TNF and IL-2, which can be completed by cell surface or intracellular assessment of activation markers expressed after T cell stimulation (e.g. CD154, CD107a). However, ICS is per se limited by the choice of the cytokines to be detected. The method is also intrinsically complex, requiring cell culture over several hours in the presence of protein transport inhibitors and time-consuming staining procedures including permeabilization/fixation steps which do not preserve cell viability. In addition, ICS has been shown to be sensitive to small variations in protocols and cell handling (3–5).

Cell surface activation induced markers (AIMs) identify reactive antigen-specific T cells independently of their cytokine production pattern, and are not restricted to certain T cell subsets or differentiation states (6). As an example, CD154 (CD40 ligand), a costimulatory molecule expressed on activated Th cells, is routinely used intracellularly or extracellularly for assessing antigen-specific CD4^+^ T cells (7–9). Further AIMs can be implemented for detection of antigen-specific CD4^+^ T cells, like combinations of CD69/CD154 or OX40/CD25, and more recently OX40/PD-L1 or OX40/CD137 (4-1BB), with stimulation times between 9 and 20 hours (h), depending on the marker combination (10–12). Since CD154 is not broadly expressed on CD8^+^ T cells, it can be replaced by CD137, OX40 or CD25 for assessing this subset (12–14). In a recent screening study of SARS-CoV-2-reactive T cells in convalescent donors, the AIM combinations CD137/OX40 and CD137/CD69 were analyzed separately for CD4^+^ and CD8^+^ T cells, respectively, after 24 h of stimulation (15). Although it would be material- (clinical samples are available in limited amount) and time-advantageous, simultaneous detection of CD4^+^ and CD8^+^ T cells with the very same AIM combination is rarely performed. Moreover, AIM expression on the cell surface of unstimulated cells is not negligible, which impairs the sensitive detection of low-frequency T cells.

ß_2_-integrins (e.g. LFA-1) are already expressed on the cell surface of resting antigen-experienced T cells in a steady-state, inactivated conformation. TCR-mediated activation induces a conformational change to an open high-affinity form and clustering of ß_2_-integrins, leading to an increase in affinity and valency which allows interaction with its ligand intercellular adhesion molecule (ICAM)-1 (16, 17). We have previously used activated ß_2_-integrins as an early cell surface activation marker that is detectable after minutes (CD8^+^ T cells) or few hours (CD4^+^ T cells) of antigen-specific stimulation. Activated ß_2_-integrins were specifically stained with fluorescent multimeric ICAM-1 (on CD8^+^ T cells) or with a conformational-specific monoclonal antibody (clone m24, on CD8^+^ and CD4^+^ T cells) (18, 19). Moreover, combination of activated ß_2_-integrin staining and intracellular cytokine staining (m24+ICS) demonstrated that m24 antibody (Ab) staining is a surrogate marker for cytokine production and allowed identification of very low frequency functional antigen-specific T cells after short *in vitro* stimulation (19).

We now introduce activated ß_2_-integrins as a cell surface activation marker that can be combined to already established AIMs for simultaneous detection of functional antigen-specific CD4^+^ and CD8^+^ T cells. First, several AIMs were tested for combination with m24 Ab staining: CD25, CD69, CD137, OX40 and CD154. Based on the following considerations (i) the expression kinetics of the different markers, ii) the feasibility of detecting simultaneously CD4^+^ and CD8^+^ T cells, and iii) the need to reduce background staining in unstimulated cells for improved detection of rare events, we propose to combine cell surface staining of ß_2_-integrins with CD137 and CD154 (m24+AIM). Detection of these markers together decreased background and correlated with intracellular cytokine staining, with higher frequencies of antigen-specific T cells being detected with m24+AIM than with m24+ICS. As an example of the application of the assay to clinical samples, we show that SARS-CoV-2 spike-specific CD4^+^ and CD8^+^ T cells can be readily detected in healthy donors after SARS-CoV-2 vaccination.

## 2 Materials and methods

### 2.1 Peripheral blood mononuclear cell isolation

Peripheral blood mononuclear cells (PBMCs) were isolated from mononuclear cell blood concentrates (cones) or Na-heparinized blood by cell density centrifugation (Lymphosep, Biowest). PBMCs were washed twice with PBS (w/o Ca^2+^/Mg^2+^) and counted with an automated cell counter (NucleoCounter, Chemometec) using Solution 18 (AO/DAPI, Chemometec). Aliquots of ~20 Mio cells/ml were frozen in 90% heat-inactivated fetal bovine serum (FBS; Capricorn Scientific) containing 10% DMSO (Merck) at -80°C and stored in liquid nitrogen until usage.

### 2.2 Study subjects and experimental settings

**Figure 1:**
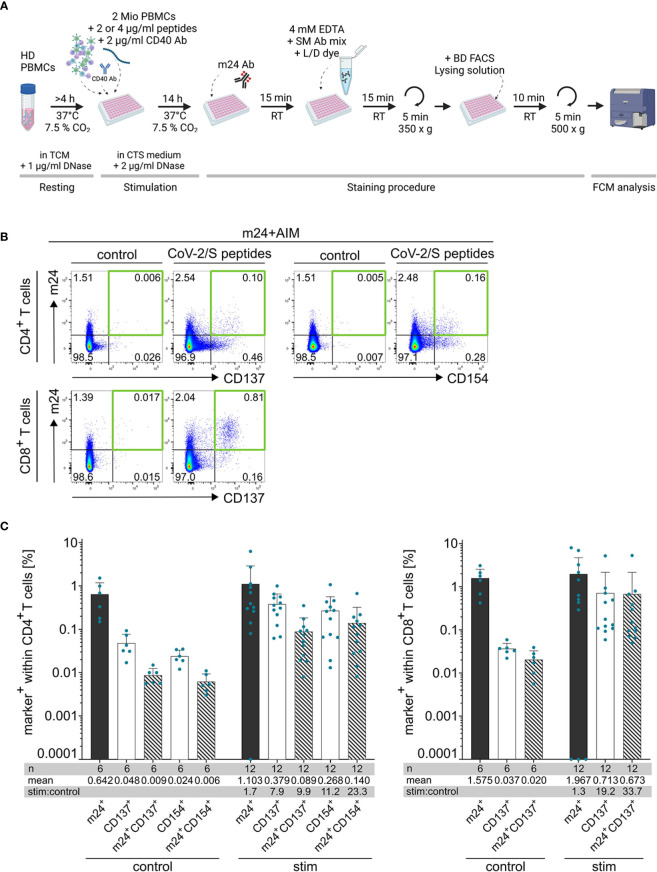
Co-staining of cell surface activated ß_2_-integrins with CD137 and CD154 (m24+AIM) allows identification of antigen-specific T cells. **(A)** Scheme of the m24+AIM protocol (created with BioRender.com). PBMCs from healthy donors (HD) are stimulated with antigenic peptides or left unstimulated for 14 h in CTS medium + DNase in the presence of CD40 Ab. Activated ß_2_-integrins are then stained with m24 Ab, followed by incubation with EDTA, surface markers (SM) and live/dead dye (L/D) before fixation and analysis by flow cytometry. **(B)** Exemplary dot plots of PBMCs from donor HD5 incubated with SARS-CoV-2/S overlapping peptides or unstimulated control. m24 Ab staining in combination with CD137 or CD154 expression on CD4^+^ T cells (top) or m24 Ab staining and CD137 expression on CD8^+^ T cells (bottom). Numbers represent frequencies within CD4^+^ or CD8^+^ T cells, the green squares mark the subsets of interest. **(C)** Mean + SD of the frequencies of cells expressing individual activation markers (unicolored bars) and combination of markers (striped bars) within CD4^+^ (left) and CD8^+^ (right) T cells. Number of donor/antigen combinations tested (n), mean and stimulated:control ratios (stim:control) are indicated. Frequencies of stimulated samples are background subtracted. Values that were 0 were set to 0.0001.

**Figure 2:**
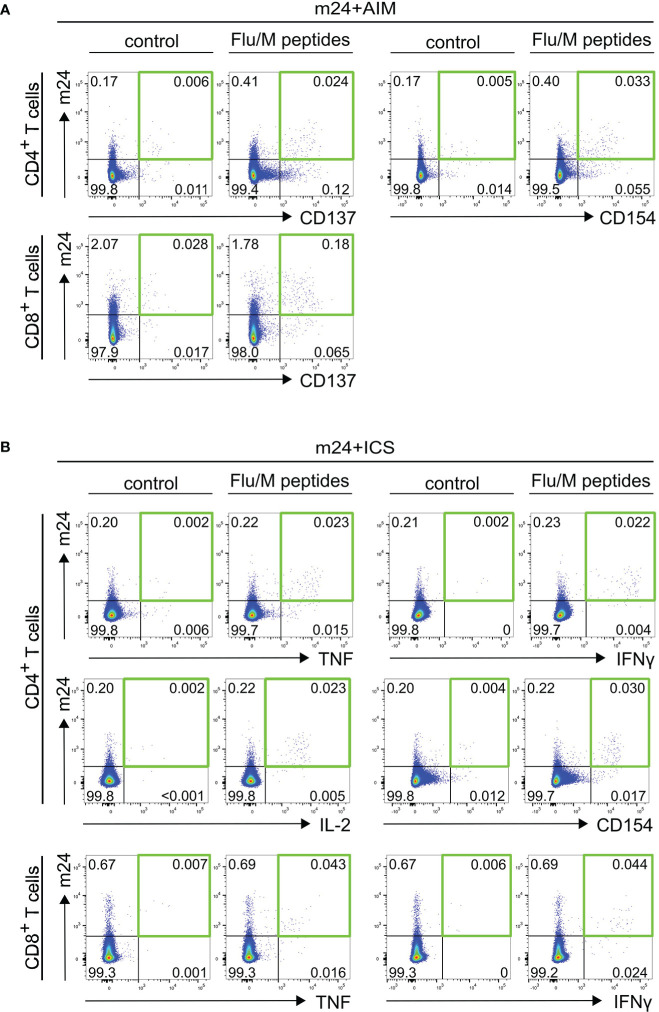
Detection of antigen-specific T cells using m24+AIM **(A)** or m24+ICS **(B)**. Exemplary dot plots of PBMCs from donor HD6 incubated with Flu/M overlapping peptides or left unstimulated for 14 h in the presence of CD40 Ab (m24+AIM) or brefeldin A and monensin (m24+ICS). **(A)** m24+AIM: m24 Ab staining in combination with CD137 or CD154 expression on CD4^+^ T cells, or m24 Ab staining and CD137 expression on CD8^+^ T cells **(B)** m24+ICS: m24 Ab staining in combination with intracellular TNF, IFNγ, IL-2 and CD154 expression for CD4^+^ T cells, or intracellular TNF and IFNγ expression for CD8^+^ T cells. Numbers represent frequencies within CD4^+^ or CD8^+^ T cells. The green squares in **(A, B)** mark the populations of interest co-expressing two markers.

**Figure 3:**
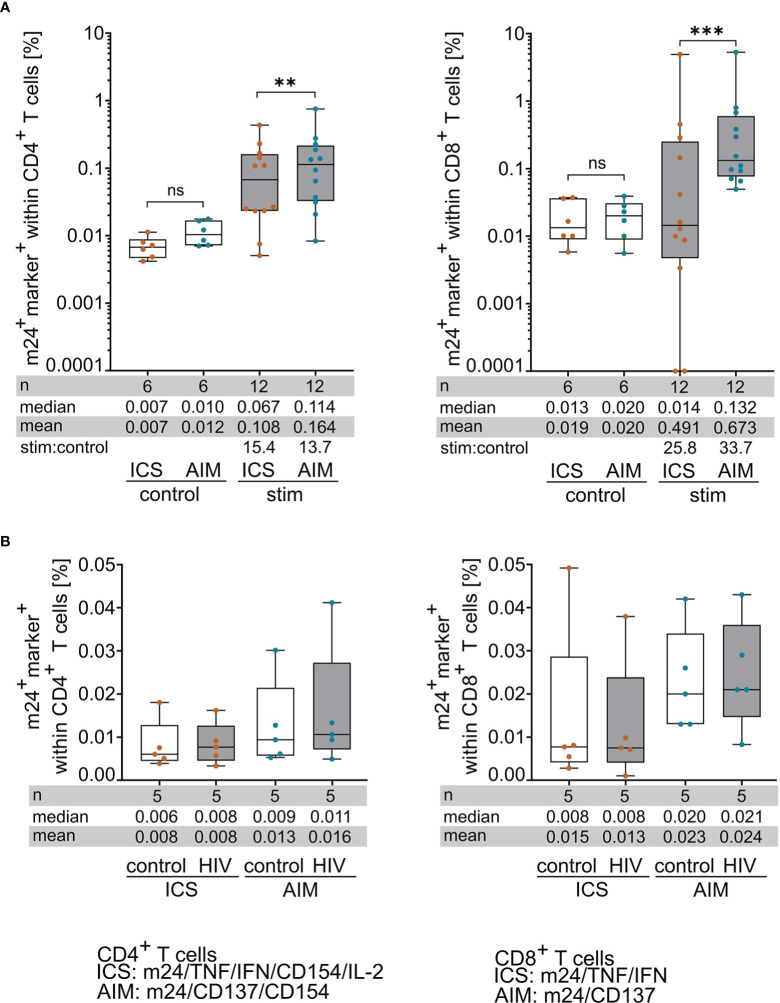
m24+AIM staining detects antigen-specific T cells with increased sensitivity compared to m24+ICS. PBMCs were incubated with antigenic peptides or left unstimulated for 14 h in the presence of CD40 Ab (m24+AIM) or brefeldin A and monensin (m24+ICS). Summed frequencies of m24^+^ and at least one additional marker^+^ T cells after Boolean gating are presented as box and whisker plots showing median, interquartile range, minimum and maximum for control and peptide-stimulated CD4^+^ (left graphs) and CD8^+^ (right graphs) T cells detected with m24+ICS or m24+AIM. Number of samples (n), median, mean and stimulated:control ratios (stim:control) are indicated. **(A)** Comparison of CD4^+^ (left) and CD8^+^ (right) T cell frequencies detected with m24+ICS or m24+AIM in unstimulated control and peptide-stimulated tests (n=6 HD, 12 donor/antigen combinations). Frequencies of stimulated samples are background subtracted. Values that were 0 were set to 0.0001. **(B)** Comparison of CD4^+^ (left) and CD8^+^ (right) T cell frequencies detected with m24+ICS or m24+AIM in control or HIV peptide-supplemented samples (n=5 HD). To compare both conditions, HIV tests were not background subtracted. Wilcoxon matched pairs, p<0.05 (** 0.002, *** < 0.001). ns, not significant.

**Figure 4:**
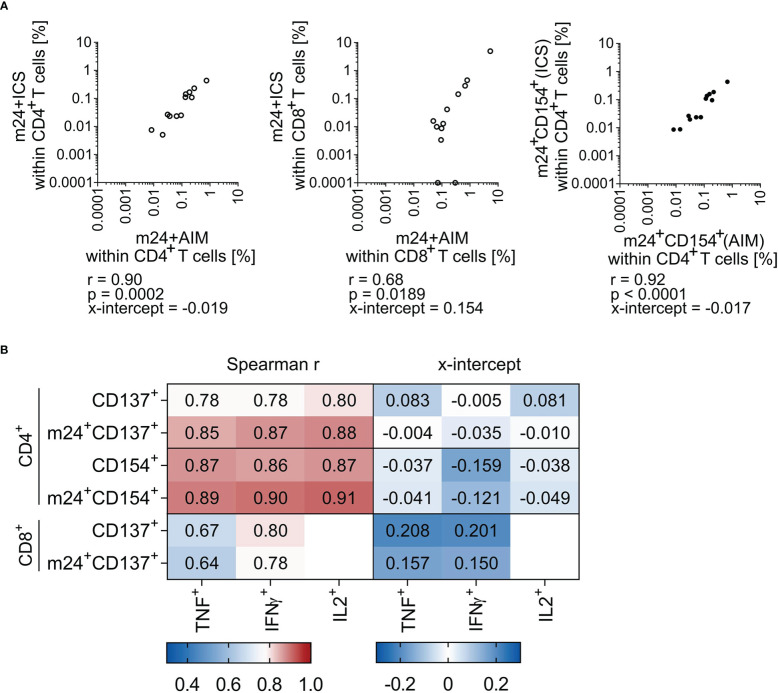
Frequencies of antigen-specific T cells assessed by m24+AIM correlate with those detected with m24+ICS. Correlation between antigen-specific T cell frequencies assessed by AIM or ICS in HD PBMCs (n=12 donor/antigen combinations). Spearman r (p<0.05) and x-intercepts are indicated. Frequencies of stimulated samples are background subtracted. Values that were 0 were set to 0.0001. **(A)** Comparison of frequencies detected with m24+AIM vs m24+ICS (summed frequencies of m24^+^ and at least one additional marker^+^ after Boolean gating) for CD4^+^ (left) and CD8^+^ (middle) T cells. Comparison of extracellular vs intracellular CD154 staining for CD4^+^ T cells (right). **(B)** Heat map of Spearman r (left) and x-intercept (right). Frequencies of antigen-specific CD4^+^ (top) and CD8^+^ (bottom) T cells detected by single AIMs or in combination with m24 Ab staining were compared to single cytokine expression. Corresponding correlation graphs are shown in **Supplementary Figure 3**.

**Figure 5:**
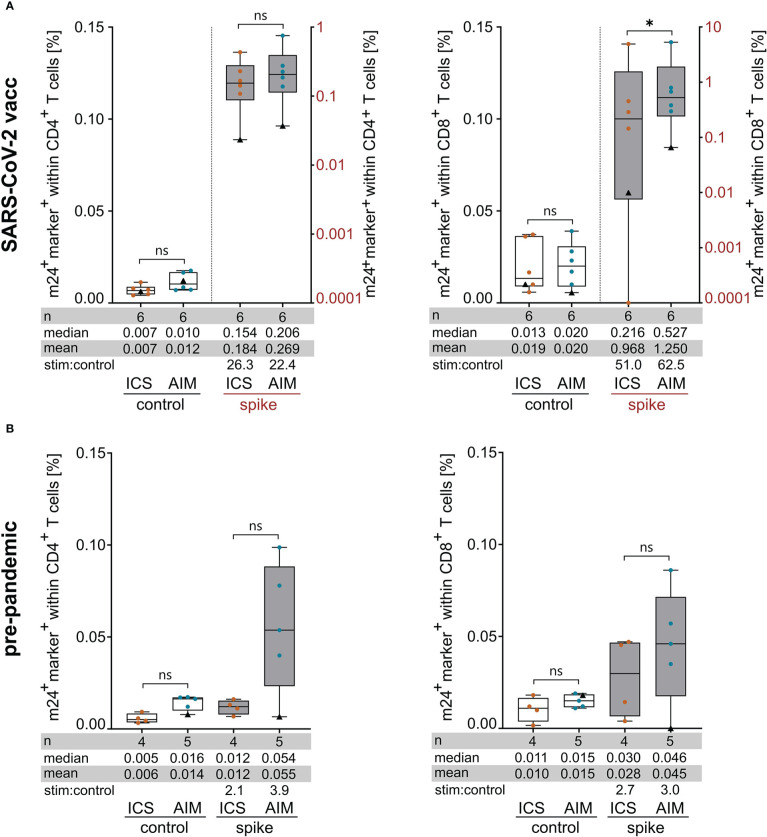
Assessment of spike-specific T cell responses with m24+ICS and m24+AIM in SARS-CoV-2 vaccinated **(A)** and pre-pandemic **(B)** healthy donors. PBMCs were incubated with SARS-CoV-2/S overlapping peptides or left unstimulated for 14 h in the presence of CD40 Ab (m24+AIM) or brefeldin A and monensin (m24+ICS). Summed frequencies of m24^+^ and at least one additional marker^+^ T cells after Boolean gating are presented as box and whisker plots showing median, interquartile range, minimum and maximum for CD4^+^ (left graphs) and CD8^+^ (right graphs) T cells. Number of donors (n), median, mean and stimulated:control ratios (stim:control) are indicated. For donor HD7 (black triangle), pre-pandemic and post-vaccination samples are shown. Frequencies of stimulated samples are background subtracted. **(A)** T cell responses in SARS-CoV-2 vaccinated donors (n=6 HD). Frequencies of marker^+^ cells in unstimulated controls (left y-axis) and after spike-peptide stimulation (right y-axis) are plotted for CD4^+^ and CD8^+^ T cells. This dataset is also part of **Figures 1**, **3** and **4**. Values that were 0 were set to 0.0001. **(B)** Frequencies of marker^+^ cells in pre-pandemic PBMCs tested with m24+AIM (n=5 HD) and m24+ICS (n=4 HD, donor HD7 symbolized by a black triangle was tested only with m24+AIM). p<0.05 (* 0.03). ns, not significant.

PBMCs from altogether 21 healthy donors (HD) were used (**Supplementary Table 1**). Expression kinetics were performed with two of them (**Supplementary Figure 1A**, HD2 and HD21). Three HLA-A*02^+^ donors with detectable Flu-, CMV- or EBV-specific CD4^+^ and CD8^+^ T cell responses in pre-screening experiments (performed with multimer stainings for CD8^+^ T cells and ICS after synthetic peptide stimulation for CD4^+^ T cells) were used for further AIM selection (**Supplementary Figures 1B, C**, HD1, 3 and 4). Three HLA-A*02^+^ and two HLA-A*02^-^ donors (HD4, HD11-14) were tested with HIV-derived peptides. Three donors were used to compare the staining procedure of m24+AIM with and without cell fixation (**Supplementary Figure 4**, HD11, HD19 and HD20). For the comparison of m24+AIM and m24+ICS assays, we collected PBMCs from six healthy donors between three to five weeks after the third SARS-CoV-2 vaccination and screened them for SARS-CoV-2-specific, as well as for Flu-specific T cell responses (HD5-10, **Figures 1B, C**, **2A, B**, **3A**, **4A, B**, **5A**). Pre-pandemic samples from five individuals collected between September 2019 and January 2020 were also tested for SARS-CoV-2-specific T cell responses (HD7, HD15-18, **Figure 5B**), one of these (HD7) was also tested after SARS-CoV-2 vaccination.

Blood collection and study were approved by the Ethics Committee of the University of Tübingen (approvals 156/2012B01 and 713/2018BO2), and all participants gave written informed consent.

### 2.3 Peptides and stimuli

For simultaneous antigen-specific stimulation of CD8^+^ and CD4^+^ T cells, synthetic HLA-class I and -class II peptides either produced in-house or purchased as 15-mer overlapping peptide pools were used (**Supplementary Table 1**).

A HLA-class II peptide mix of nine described epitopes derived from various proteins of CMV (n = 2), EBV (n = 6), and Flu (n = 1) viruses (Mix-II) together with the HLA-A*02 binding GLCTLVAML epitope from EBV BMLF1 protein, aa 259-267 (EBV/GLC) was used. For screening HIV-specificities, the epitopes ILKEPVHGV (HIV RT, aa 476-484, HLA-A*02 binder) and YVDRFYKTLRAEQASQEV (HIV Gag, aa 164-181, HLA-DRB1 binder) were pooled (HIV I+II). All in-house synthetized peptides were dissolved in 10% DMSO/H_2_O and stored at -80°C at a concentration of 1 mg/ml. Pools of 15-mer peptides overlapping by eleven amino acids and spanning the entire CMV/pp65 (CMV/pp65 peptides) and Flu/matrix proteins (Flu/M peptides) were purchased from JPT Peptide Technologies, those spanning the entire SARS-CoV-2/spike (CoV-2/S peptides) from Miltenyi Biotec. Peptide pools from JPT were dissolved to a concentration of 500 µg/ml in 100% DMSO, while the spike peptide pool was dissolved to a concentration of 100 µg/ml in 20% DMSO/H_2_O, aliquoted and stored at -80°C. As positive control, cells were stimulated with Staphylococcal enterotoxin B (SEB; Sigma-Aldrich). SEB was dissolved in PBS w/o Ca^2+^/Mg^2+^ and stored at -20°C in 1 mg/ml aliquots. Unstimulated control samples were supplemented with a final concentration of DMSO identical to that used in the stimulated tests.

### 2.4 Thawing and resting of PBMCs

Cryopreserved PBMCs were thawed in IMDM (Gibco) containing 2.5% heat-inactivated (hi) human serum (HS; Capricorn Scientific), 100 U/ml penicillin/100 µg/ml streptomycin (1X P/S, Sigma-Aldrich) and 50 µM β-mercaptoethanol (ß-ME; Merck). After washing once (300 x g, 5 min, room temperature (RT)) with serum free IMDM medium (IMDM, P/S, 50 µM ß-ME), cells were counted and rested for 4 to 6 h in T cell medium (TCM; IMDM, 10% HS hi, 1X P/S, 50 µM ß-ME) containing 1 µg/ml DNase I (Sigma-Aldrich) at a concentration of 2-3 Mio cells/ml and not more than 20 Mio cells per 50 ml Falcon tube at 37°C and 7.5% CO_2_.

### 2.5 Kinetics for detection of cell surface AIMs CD25, CD69, CD137, OX40 and CD154

After resting, cells were washed (300 x g, 5min, RT) with serum free IMDM medium, counted, and resuspended in the appropriate volume of TCM for stimulation. For cell surface staining of CD25, CD69, CD137 and OX40, 2 Mio PBMCs were incubated with 10 µg/mL SEB or left unstimulated in a final volume of 150 µl in sterile 96 well U-bottom culture plates (Greiner Bio-One) for 4, 6, 14 and 24 h at 37°C and 7.5% CO_2_. After stimulation, 50 µl FACS buffer (PBS, 2% FBS hi, 2mM EDTA and 0.02% sodium azide) was added to the wells, samples were centrifuged (380 x g, 5 min, RT), washed with 150 µl FACS buffer and stained with a mix of live/dead dye (Zombie aqua fixable dye, Biolegend), CD4-FITC (in-house production), CD8-AF700, CD25-PE-Cy7, CD69-BV421, CD137-PE-Dazzle594 and OX40-APC antibodies (Abs) in 50 µl FACS buffer. For extracellular detection of CD154, cells must be stimulated in the presence of a purified CD40 Ab to prevent cell surface downregulation and internalization of CD154 upon ligand binding (6, 8, 20). 2 Mio PBMCs were incubated with 10 µg/mL SEB or left unstimulated in the presence of 2 µg/ml CD40 Ab (pure-functional grade, clone HB14, Miltenyi Biotec) in a final volume of 150 µl in sterile 96 well U-bottom culture plates for 4, 6, 14 and 24 h at 37°C and 7.5% CO_2_. After stimulation, 50 µl FACS buffer was added, samples were centrifuged, washed with 150 µl FACS buffer and stained with a mix of Zombie aqua, CD4-FITC, CD8-AF700 and CD154-APC Abs in 50 µl FACS buffer. Stainings were performed for 20 min at 4°C in the dark. Cells were washed twice with 150 µl FACS buffer, resuspended in FACS buffer and analyzed on the same day. All Abs were purchased from Biolegend, if not stated differently, and were used at recommended or pre-tested dilutions.

### 2.6 Stimulation of antigen-specific CD4^+^ and CD8^+^ T cells

After resting, cells were washed twice (300 x g, 5min, RT) with CTS medium (CTS OpTmizer T-Cell Expansion SFM containing supplement (Gibco), 2 mM L-Glutamine (Sigma-Aldrich) and 1X P/S) and counted before resuspending in CTS medium + 2 µg/ml DNase I for stimulation (optimized medium conditions for T cell recovery and m24 Ab staining). 2 Mio PBMCs were stimulated in a final volume of 200 µl in sterile 96 well U-bottom culture plates (Greiner Bio-One) for 14 h at 37°C and 7.5% CO_2_ in the presence of 10 µg/ml brefeldin A (Sigma-Aldrich) and Golgi Stop (1:1500, BD Biosciences) for intracellular marker staining (m24+ICS), or in the presence of 2 µg/ml CD40 Ab (pure-functional grade, clone HB14, Miltenyi Biotec) for staining of cell surface activation induced marker (m24+AIM). Purchased overlapping peptide pools or in-house synthetized peptides were used at a final concentration of 2 µg/ml or 4 µg/ml per peptide, respectively.

### 2.7 m24 Ab staining combined to intracellular marker staining

After stimulation, samples were incubated for 4 min at RT while removing 100 µl of the supernatant thus leaving 100 µl of the stimulation volume without disturbing the cell pellet. Activated ß_2_-integrins were stained with a PE-labeled monoclonal Ab (m24 Ab clone, 1:100) and live/dead dye Zombie aqua for 15 min. 100 mM EDTA diluted in PBS was added to a final concentration of 4 mM EDTA and incubated for 10 min. Samples were centrifuged (380 x g, 5 min), fixed with 200 µl 1X FACS-Lysing solution (BD Biosciences) for 10 min, and washed with PBS/0.5% BSA/0.1% sodium azide (500 x g, 5 min). All following centrifugation steps were performed at 500 x g (5 min) and all washing steps, as well as Ab dilutions, were in PBS/0.5% BSA/0.1% sodium azide. CD3-PerCP-Cy5.5, CD4-BV421, and CD8-APC-Cy7 Abs were added in 50 µl volume for 15 min. Cells were washed by adding 150 µl wash buffer, followed by permeabilization for 20 min with 200 µL 1X FACS-Permeabilizing solution 2 (BD Biosciences). Samples were centrifuged and stained for intracellular markers with TNF-BV605, IFNγ-FITC, IL-2-PE-Cy7 and CD154-APC Abs in 50 µl volume for 30 min. After two more washing steps, cells were resuspended in FACS buffer and analyzed on the same day. All Abs were purchased from Biolegend and pre-titrated. Incubation, staining and centrifugation steps were performed at RT in the dark.

### 2.8 m24 Ab staining combined to cell surface activation marker staining

After stimulation, samples were incubated for 4 min at RT while removing 100 µl of the supernatant thus leaving 100 µl of the stimulation volume without disturbing the cell pellet. Activated ß_2_-integrins were stained with PE-labeled m24 Ab (1:100) for 15 min. A mix of live/dead dye Zombie aqua, CD3-PerCP-Cy5.5, CD4-BV421, CD8-APC-Cy7, CD154-APC, CD137-PE-Dazzle594 Abs diluted in PBS/0.5% BSA/0.1% sodium azide supplemented with 4 mM EDTA was added directly into the remaining stimulation volume (final volume 125 µl) and incubated for 15 min. When CD69 was stained (**Supplementary Figure 1C**, HD4), Zombie aqua, CD3-PerCP-Cy5.5, CD4-APC-Cy7 (BD Biosciences), CD8-AF700, CD154-APC, CD137-PE-Dazzle594 and CD69-BV421 Abs were used. Afterwards, samples were centrifuged (380 x g, 5 min), fixed with 200 µl 1X FACS-Lysing solution (BD Biosciences) for 10 min and washed with PBS/0.5% BSA/0.1% sodium azide (500 x g, 5 min). Cells were resuspended in FACS buffer and analyzed on the same day. All Abs were purchased from Biolegend, if not stated differently, and pre-titrated. Incubation, staining and centrifugation steps were performed at RT in the dark.

### 2.9 Flow cytometry analysis

Samples were acquired on a BD LSRFortessa with the Diva software V.6 (between 450,000 and 1,000,000 events were recorded) and analyzed with Flow Jo 10.6.2 (BD Biosciences). Gating strategy is shown in **Supplementary Figure 2A**. Results are presented as percent of cells within the parent CD4^+^ or CD8^+^ living CD3^+^ lymphocyte population. To compare the combination of m24 Ab and cell surface AIM staining (m24+AIM) with the combination of m24 Ab and intracellular activation marker staining (m24+ICS), frequencies of m24^+^ and at least one additional marker^+^ T cells after Boolean gating (21) were summed up (**Supplementary Figure 1D** and **Figures 3A, B**, **4A**, **5A, B**). Depending on the cell type (CD4^+^ or CD8^+^) and the staining procedure (m24+AIM or m24+ICS), different marker combinations were considered: m24 Ab, CD154 and CD137 or m24 Ab, TNF, IFNγ, IL-2 and CD154 for CD4^+^ T cells; m24 Ab and CD137 or m24 Ab, TNF and IFNγ for CD8^+^ T cells. For assessing antigen-specific T cells, frequencies of marker^+^ cells detected in the unstimulated control samples were subtracted from the frequencies of marker^+^ cells in the stimulated samples (background subtracted). Stimulated:control ratios were calculated by dividing the mean frequency of antigen-specific T cells by the mean frequency of the corresponding unstimulated control samples (**Figures 1C**, **3A**, **5A, B**)

### 2.10 Graphs and statistical analysis

Graphs and statistical analyses were prepared with Prism (GraphPad, version 9.4.0). For comparison of m24+AIM and m24+ICS datapoints, a Wilcoxon matched pairs test was performed (significant if p<0.05, **Figures 3A, B**, **5A, B**). Spearman correlation analyses and simple linear regressions were used to determine Spearman r and x-intercepts (**Figures 4A, B** and **Supplementary Figure 3**), respectively. x-intercept gives information on the closeness of the results between the two parameters or assays compared. If data was plotted on a logarithmic scale, values that were 0 were set to 0.0001 (**Figures 1C**, **3A**, **4A**, **5A** and **Supplementary Figure 3**).

## 3 Results

### 3.1 Combination of cell surface staining of activated ß_2_-integrins and AIMs CD137 and CD154 is optimal for detection of antigen-specific CD4^+^ and CD8^+^ T cells

To determine the optimal conditions for co-staining activated ß_2_-integrins with other established AIMs, we first examined which markers can be detected simultaneously. The expression kinetics of various AIMs, namely CD25, CD69, CD137, OX40 and CD154, were assessed in PBMCs from two healthy donors (HD PBMCs) stimulated with SEB for 4, 6, 14 and 24 h. CD25 expression was almost not detectable at 4 and 6 h and reached maximum at 24 h on both CD4^+^ and CD8^+^ T cells. OX40 shows very low expression on CD8^+^ T cells and low expression on CD4^+^ T cells after 4 and 6 h of stimulation, with maximum also reached at 24 h. Both markers are therefore not favorable for a fast read out (**Supplementary Figure 1A**). CD69 and CD137 were upregulated on both CD4^+^ and CD8^+^ T cells; CD69 expression was evident already at 4 h of stimulation and remained stable up to 24 h, while CD137 expression peaked between 6 and 14 h of stimulation (**Supplementary Figure 1A**). In accordance with previous reports (8, 9), CD154 was detectable on CD4^+^ T cells, with maximum expression reached at 4 h and maintained up to 14 h after stimulation (**Supplementary Figure 1A**), but was poorly expressed on CD8^+^ T cells (**Supplementary Figures 1A, C, D**). Hence, expression of CD69, CD137 and CD154 can be optimally detected after overnight (14 h) T cell activation.

We previously showed that ß_2_-integrin activation is an early event (approx. 5-15 min and 4-6 h for CD8^+^ and CD4^+^ T cells, respectively) following antigen-specific *in vitro* stimulation (18, 19). To combine this marker with further AIMs, we verified that ß_2_-integrin activation was detectable after 14 h of stimulation (**Supplementary Figure 1B**). For CD4^+^ T cells, frequency of m24^+^ cells increased between 4 and 14 h of stimulation (e.g. 0.58% to 0.70% for HD1), with minor changes in the expression within the unstimulated control (e.g. 0.063% to 0.075% for HD1, **Supplementary Figure 1B**, top). For CD8^+^ T cells, frequency of m24^+^ cells decreased between 4 and 14 h of stimulation, however frequency in the unstimulated control also decreased, which led to an increased signal-to-noise (stimulated:control) ratio (**Supplementary Figure 1B**, e.g. for HD1 stimulated:control ratios 5.8 (4 h) and 8.3 (14 h)).

Combination of m24 Ab staining with detection of CD69, CD137 and CD154 after 14 h of stimulation in the presence of CD40 Ab (to prevent downregulation of CD154 upon ligand binding) was tested in four donors that were previously screened for antigen reactivities, one representative donor is presented in **Supplementary Figure 1C**. Staining of CD69 showed very high background in unstimulated cells, and a distinct population in the antigen-stimulated sample was difficult to identify (**Supplementary Figures 1A, C** left panel), hence, this marker was not kept for further testing. In contrast, combination of m24 Ab staining with detection of CD137 (for CD8^+^ and CD4^+^ T cells) and CD154 (for CD4^+^ T cells) allowed clear identification of antigen-reacting T cells (**Supplementary Figure 1C**, middle and right panels). Based on these results our final protocol combines m24 Ab staining with AIMs CD137 for CD8^+^ T cells or CD137 and CD154 for CD4^+^ T cells (m24+AIM; **Figure 1A** for schematic overview). The gating strategy adopted for the analysis is presented in **Supplementary Figure 2A** and representative dot plots are shown in **Figure 1B**.

While analysis of single markers showed notable background staining in unstimulated control cells, the combination of CD137 or CD154 detection with m24 Ab staining was sufficient to decrease the background (to <0.01% cells and 0.02% in mean for CD4^+^ and CD8^+^ T cells, respectively; **Figures 1B, C**). In the stimulated samples, the combination of markers also showed a decline in the frequencies of CD4^+^ responding T cells compared to single marker stainings, but not as consequent as for the controls (**Figure 1C**). Importantly, the stimulated:control ratio was greatly improved when combining m24 Ab with CD137 or CD154 staining for CD4^+^ T cells, or m24 Ab and CD137 for CD8^+^ T cells (**Figure 1C**, bottom tables). Hence, combination of m24 Ab staining with AIMs CD137 and CD154 improved the signal-to-noise ratio and thereby gives the possibility to detect very low frequencies of antigen-specific CD4^+^ and CD8^+^ T cells.

### 3.2 Combined cell surface staining of activated ß_2_-integrins with CD137 and CD154 is more sensitive than intracellular marker staining to identify functional antigen-specific T cells

Activated ß_2_-integrins are expressed by the majority of cytokine-expressing T cells after antigen-specific stimulation (18, 19). We therefore compared the frequencies of antigen-specific T cells detected by combination of activated ß_2_-integrins and cell surface activation marker staining (m24+AIM) with that detected by combination of activated ß_2_-integrin and intracellular activation marker staining (m24+ICS), which is a broadly accepted read out to detect functional T cells. Since cell surface expression of activation markers is inhibited in the presence of brefeldin A and monensin [(7) and data not shown], all markers could not be assessed simultaneously, and two independent tests were performed in parallel (m24+AIM vs m24+ICS). For CD4^+^ T cells, the combinations of m24 Ab with CD154 or CD137 vs m24 Ab with TNF, IFNγ, IL-2 or intracellular CD154 were assessed. For CD8^+^ T cells, the combination of m24 Ab and CD137 was compared to that of m24 Ab and TNF or IFNγ. PBMCs from six donors (HD5-HD10) were tested for SARS-CoV-2/S- and Flu/M-reactive T cell responses (n=12 donor/antigen combinations). Exemplary dot plots of m24+AIM and m24+ICS stainings of CD4^+^ and CD8^+^ T cells stimulated with Flu/M overlapping peptides are shown in **Figures 2A, B** for donor HD6. An additional example for m24+AIM and m24+ICS stimulated with SARS-CoV-2/S peptides is presented for donor HD5 in **Figure 1B** and **Supplementary Figure 2B**, respectively.

The summed frequencies of m24^+^ and at least one additional marker^+^ T cells after Boolean gating were calculated for each donor/antigen combination (n=12) detected with either m24+AIM or m24+ICS. **Figure 3A** displays the results obtained for unstimulated controls and antigen-specific stimulations of CD4^+^ (left graph) and CD8^+^ (right graph) T cells. The frequencies of reactive T cells in unstimulated control samples were slightly higher in both T cell subsets for m24+AIM compared to m24+ICS, but these differences were not significant (n=6, **Figure 3A**). The frequency of antigen-specific T cell responses was however significantly increased in CD4^+^ T cells (p=0.001) and even more pronounced in CD8^+^ T cells (p<0.001) for m24+AIM compared to m24+ICS (n=12, **Figure 3A**).

To rule out that non-antigen specific T cells can express activated ß_2_-integrins and AIMs in our experimental setting (which could explain the difference in the observed frequencies after m24+AIM vs m24+ICS staining), we incubated HD PBMCs (n=5, n=2 HLA-A*02^-^ and n=3 HLA-A*02^+^ donors) with a mix of two known HIV-derived epitopes, one for HLA-class I (HLA-A*02) and one for HLA-class II (HLA-DRB1), against which we did not expect to observe any reactive T cells. Again, the summed frequencies of m24^+^ and at least one additional marker^+^ T cells after Boolean gating were calculated for each donor. These frequencies were neither significantly different between the HIV- and the corresponding control stimulations nor between the m24+AIM vs the m24+ICS assays, both for the CD4^+^ (left graph) and CD8^+^ (right graph) T cell subsets (**Figure 3B**). Altogether, these results suggest that T cells detected with the m24+AIM assay are not activated by our experimental conditions but are indeed antigen-specific, and that the assay is able to detect higher frequencies of antigen-reactive T cells as compared to m24+ICS staining.

For in depth comparison of both assays (m24+AIM vs m24+ICS), we additionally performed correlation analyses and calculated x-intercepts (n=12 donor/antigen combinations from **Figure 3A**). Combination of m24 Ab, CD137 and CD154 highly correlated with combination of m24 Ab, IFNγ, TNF, IL-2 and CD154 for CD4^+^ T cells with a Spearman r of 0.90 (p=0.0002, **Figure 4A** left graph). The two assays (m24 Ab and CD137 vs m24 Ab, IFNγ and TNF) also correlated for CD8^+^ T cells, but with a lower Spearman r of 0.68 (p=0.0189, **Figure 4A** middle graph). The only marker that could be directly compared in intracellular (m24+ICS) vs extracellular (m24+AIM) staining was CD154 on CD4^+^ T cells, with a notable Spearman r of 0.92 (p<0.0001, **Figure 4A** right graph). For CD4^+^ T cells, x-intercepts were -0.019 (m24+AIM vs m24+ICS) and -0.017 (extracellular vs intracellular CD154 stainings) (**Figure 4A**). For CD8^+^ T cells, a higher x-intercept value was observed (0.154, **Figure 4A**), reflecting the increased frequencies of antigen-specific T cells detected with m24+AIM compared to m24+ICS (**Figure 3A**).

To verify the utility of combining AIM markers with m24 Ab staining, we finally performed correlations with single cytokine expression (frequencies of cells detected by CD154 or CD137 expression alone or combined to m24 Ab staining vs TNF, IFNγ or IL-2 production, see **Figure 4B** and corresponding graphs **Supplementary Figure 3**). For CD4^+^ T cells, CD137 expression alone showed a weaker correlation with cytokine production as compared to CD137 combined to m24 Ab staining, CD154 expression alone and CD154 combined to m24 Ab staining (Spearman r, **Figure 4B** left panel). For example, Spearman r were 0.78 vs 0.85 for TNF detection with CD137 alone vs CD137 combined to m24 Ab staining, respectively. For CD8^+^ T cells, CD137 alone or in combination with m24 Ab showed similar correlations with both TNF and IFNγ (Spearman r 0.64-0.80, **Figure 4B** left panel), however, x-intercepts were improved for CD137 expression combined to m24 Ab staining vs solely CD137 (**Figure 4B** right panel). Finally, detection of CD154 alone or together with m24 Ab staining appears equally good for identifying antigen-specific CD4^+^ T cells (Spearman r and x-intercepts). Altogether, m24+AIM correlates with m24+ICS and the combination of m24 Ab staining with detection of CD154 or CD137 showed improved correlations with cytokines as compared to CD137 alone.

In conclusion, m24+AIM surface staining highly correlates with intracellular cytokine production, while detecting functional antigen-specific CD4^+^ and CD8^+^ T cells with an increased sensitivity.

### 3.3 Sensitive detection of SARS-CoV-2 spike-specific T cell responses in SARS-CoV-2 vaccinated and pre-pandemic healthy donors

Finally, we compared the two assays m24+AIM and m24+ICS for their ability to detect antigen-specific T cell responses in a clinically relevant setting. For this, the T cell responses obtained for SARS-CoV-2 vaccinated donors after stimulation with overlapping spike peptides (data already included in previous sections together with other antigen stimulations, i.e. in **Figures 1**, **3**, **4**) were compared to those detected in PBMCs isolated before the SARS-CoV-2 outbreak (**Figures 5A, B**; CD4^+^ and CD8^+^ T cell responses on left and right graphs, respectively). We detected spike-specific CD4^+^ T cell responses with both assays in 6 of 6 vaccinees, while for CD8^+^ T cells, T cell reactivity was observed in 5 of 6 donors with m24+ICS, and in 6 of 6 donors with m24+AIM (**Figure 5A**). In line with published results (15, 22, 23), spike-specific CD4^+^ and/or CD8^+^ T cell responses could also be detected in 4 of 5 pre-pandemic donors, suggesting T cell cross-reactivity between CoV-2 and other viruses from the SARS family. Altogether, the mean and median frequencies of specific T cells detected with the m24+AIM assay were equivalent or even higher (**Figure 5A**, upper right panel for CD8^+^ T cell responses in vaccinees) than those detected with the m24+ICS combination. In addition, the stimulated:control ratio was elevated in 3 out of 4 cases, confirming our previous results.

## 4 Discussion

We describe a protocol for simultaneous and sensitive assessment of functional antigen-specific CD4^+^ and CD8^+^ T cells using a combination of solely cell surface activation markers, namely activated ß_2_-integrins, CD137 and CD154. Several features of activated ß_2_-integrins make them an attractive new activation induced marker to detect antigen-specific T cells, i.e. their expression on T cells at various differentiation stages, high correlation with cytokine production, and the possibility to preserve cell viability during labelling (18, 19).We have previously observed that detection of activated ß_2_-integrins showed some background staining (19), which is also the case for other established cell surface AIMs. Hence, we reasoned that the combination of these cell surface activation markers will decrease unspecific signal and lower the threshold of detection, as we demonstrated for the combination of activated ß_2_-integrins with intracellular cytokines (19).

We found that m24 Ab staining could be combined with AIMs CD137 and CD154 after a relatively short stimulation time of 14 hours (i.e. overnight). This time was compatible with the expression kinetics of these two receptors (**Supplementary Figure 1**) and in accordance with previous reports from others (6). Other described AIMs require longer stimulation times of 18 to 20 h for peak expression [e.g. OX40, CD25 (11, 12)], or show a very high background staining in unstimulated cells [e.g. CD69, **Supplementary Figure 1** and (6)]. Overall, combined staining of activated ß_2_-integrins (using m24 Ab) and CD137 decreased background of unstimulated controls and improved signal-to-noise ratio for CD8^+^ T cells. The same was seen for CD4^+^ T cells with combination of m24 Ab staining and detection of CD137 or CD154.

Interestingly, not all AIM^+^ T cells co-stained for activated ß_2_-integrins. This was especially true for the CD4^+^ subset, for which we repeatedly detected CD137- or CD154-expressing CD4^+^ T cells that were not stained with m24 Ab (**Figure 1C**). For the CD8^+^ subset however, the vast majority of antigen-specific CD137-expressing cells stained positive for activated ß_2_-integrins (>80%, **Figure 1C**). Additionally, the majority of m24^+^CD137^+^ CD4^+^ T cells also expressed CD154 (74%) and inversely (66%, data not shown). Hence, the three AIMs identify overlapping, but not fully identical CD4^+^ T cell subsets, as previously reported using AIM assays combining CD69/CD154 and OX40/CD25 (11).

To investigate the association between cell surface activation marker expression (m24+AIM) and cytokine production (m24+ICS), we performed both tests in parallel after peptide stimulation. A first finding was that the overall frequency of antigen-specific T cells detected with m24+AIM was significantly higher than that detected with m24+ICS (in mean 1.5-fold for CD4^+^ and 1.4-fold for CD8^+^ T cells, **Figure 3A**). In some cases, we could even reveal T cell responses with m24+AIM that were not detectable with m24+ICS (**Figures 3A**, **5A, B**). In contrast, addition of HIV peptides during the culture did not lead to an increase in the frequencies of responding T cells in the m24+AIM vs m24+ICS assays (**Figure 3B**). This suggests that cells revealed by cell surface staining are antigen-specific, but do not produce detectable amount of the cytokines (IFNγ, TNF and IL-2) tested. In line with these findings, AIM assays have previously been reported to identify higher frequencies of antigen-specific T cells compared to cytokine-based assays (12, 24). It is important to note that, although bystander activation is unlikely (11), it cannot be ruled out in our system. A second observation made by comparing m24+AIM and m24+ICS was an excellent correlation (Spearman r approx. 0.9) of cell surface staining with cytokine production for CD4^+^ T cells. Notably, the combination of m24 Ab staining together with CD137 or CD154 showed improved correlations compared to the detection of CD137 alone (but not CD154 alone). For CD8^+^ T cells, the correlation was weaker (Spearman r approx. 0.7) than for CD4^+^ T cells, however x-intercept was improved when m24 Ab and CD137 were combined compared to CD137 alone.

Considering these results together, i.e. the frequencies of detected cells, the signal-to-noise ratio and the correlation with cytokine production, staining of activated ß_2_-integrins, CD137 and CD154 allows optimized and simultaneous detection of functional antigen-specific CD4^+^ and CD8^+^ T cells. With this setting, frequencies of antigen-specific cells below 0.01% and 0.05% were detected for CD4^+^ and CD8^+^ T cells, respectively. Hence, the assay is highly sensitive, although spiking experiments will be needed to determine the lowest limits of detection and quantification more precisely. Moreover, the assay is simpler than the ICS method, since it doesn’t require permeabilization, resulting in less washing and centrifugation steps. As an exemplary application, we used our new method for detection of antigen-specific T cells in a cohort of SARS-CoV-2 vaccinated and unexposed donors. Spike-specific CD4^+^ and CD8^+^ T cells were detected in all SARS-CoV-2 vaccinated individuals with m24+AIM, including one CD8^+^ T cell response that was not detectable with m24+ICS (**Figure 5A**). Additionally, we observed spike-specific T cell responses in pre-pandemic donors, and again, one of the CD8^+^ T cell responses was detected with m24+AIM and not with m24+ICS (**Figure 5B**). This underlines that not all antigen-specific T cells are necessarily expressing cytokines. Hence, cell surface staining should allow for a fast and easy detection of SARS-CoV-2-specific T cell responses in convalescent and/or vaccinated individuals.

Compared to our previously published work (19), we have further optimized the protocol of activated ß_2_-integrin staining using m24 Ab for cryopreserved PBMC samples and for usage of 96 well plates, which is critical for high-throughput screening of clinical samples. Additionally, the use of serum-free CTS medium containing DNase I for stimulation was superior to a human serum-supplemented medium by reducing the background signal within unstimulated controls (comparison not shown). The protocol described in this work makes usage of FACS-Lysing solution (BD) to fix the cells after staining which preserves the activated conformation of ß_2_-integrins. However, comparison experiments show that the assay can also be performed without fixation (**Supplementary Figure 4**), which would allow to maintain cell viability for downstream analysis, similarly to what we previously showed for CD8^+^ T cells using mICAM-1 multimers (18).

In conclusion, we introduce activated ß_2_-integrins as an additional early detectable cell surface AIM applicable for detection of CD4^+^ and CD8^+^ T cells in combination with already established AIMs CD137 and CD154. Our new assay allows assessment of functional antigen-specific CD4^+^ and CD8^+^ T cells with an increased sensitivity compared to intracellular cytokine detection. It could therefore be applied for T cell epitope screening projects and for the monitoring of low frequency antigen-specific T cell responses in the context of pathogen infection, autoimmunity and cancer.

## Data availability statement

The raw data supporting the conclusions of this article will be made available by the authors, without undue reservation.

## Ethics statement

Blood collection and study were approved by the Ethics Committee of the University of Tübingen, and all participants gave written informed consent.

## Author contributions

AS, AM, H-GR, JB, SD, and CG conceived the study and designed the experiments; AS, FK and SD performed the experiments; AS and AM performed the data analysis; AS prepared the figures; AS, CG, and SD wrote the manuscript with contribution from all authors; CG and SD jointly supervised the study. All authors contributed to the article and approved the submitted version.
