# Supplementary material for: Staining of activated ß2-integrins in combination with CD137 and CD154 for sensitive identification of functional antigen-specific CD4+ and CD8+ T cells
Source: Front Immunol. 2023 Jan 19;13:1107366. doi: 10.3389/fimmu.2022.1107366 (PMC9892897; doi:10.3389/fimmu.2022.1107366)
Supplement: Supplementary file 1 [file DataSheet_1.pdf]

Supplementary Material

Supplementary Table 1: Donor ID, sex and age, collection timepoint, stimulus and corresponding figures in which the donors are presented

Donor	Sex/ Age	Collection timepoint	Stimulus	Corresponding figures
HD1 (A*02$^+$)	m/45	Apr 2021	Mix-II+EBV/GLC, CMV/pp65 peptides	S1B
HD2 (A*02$^+$)	m/55	Sep 2022	SEB	S1A
HD3 (A*02$^+$)	m/47	Oct 2020	Mix-II+EBV/GLC	S1B
HD4 (A*02$^+$)	m/34	Sep 2021	Flu/M peptides, HIV I+II	S1C, 3B
HD5	f/29	Jan 2022	CoV-2/S peptides, Flu/M peptides	S1D, 1B, 1C, 3A, 4A, 4B, S2A, S2B, S3, 5A
HD6	f/29	Jan 2022	CoV-2/S peptides, Flu/M peptides	S1D, 1C, 2A, 2B, 3A, 4A, 4B, S3, 5A
HD7	f/54	Dec 2021	CoV-2/S peptides, Flu/M peptides	S1D, 1C, 3A, 4A, 4B, S3, 5A
HD8	f/27	Dec 2021	CoV-2/S peptides, Flu/M peptides	S1D, 1C, 3A, 4A, 4B, S3, 5A
HD9	f/26	Jan 2022	CoV-2/S peptides, Flu/M peptides	S1D, 1C, 3A, 4A, 4B, S3, 5A
HD10	f/22	Jan 2022	CoV-2/S peptides, Flu/M peptides	S1D, 1C, 3A, 4A, 4B, S3, 5A
HD11 (A*02$^+$)	m/65	Nov 2021	HIV I+II, CMV/pp65 peptides	3B, S4
HD12 (A*02$^+$)	m/57	Jul 2020	HIV I+II	3B
HD13 (A*02$^-$)	m/50	Jul 2020	HIV I+II	3B
HD14 (A*02$^-$)	m/54	Jul 2020	HIV I+II	3B
HD7	f/54	Jan 2020	CoV-2/S peptides	5B
HD15 (A*02$^+$)	m/29	Sep 2019	CoV-2/S peptides	5B
HD16 (A*02$^+$)	m/52	Oct 2019	CoV-2/S peptides	5B
HD17 (A*02$^+$)	m/49	Oct 2019	CoV-2/S peptides	5B
HD18 (A*02$^+$)	m/28	Oct 2019	CoV-2/S peptides	5B
HD19 (A*02$^+$)	unknown	Jan 2021	CMV/pp65 peptides	S4
HD20 (A*02$^+$)	f/25	May 2021	CMV/pp65 peptides	S4
HD21 (A*02$^-$)	m/38	Sep 2022	SEB	S1A

HD: healthy donor; m/f: male/female; SEB: Staphylococcus enterotoxin B; Mix-II+EBV/GLC: HLA-class II epitopes and GLCTLVAML HLA-A*0201 epitope from EBV BMLF1; CMV/pp65: overlapping peptides from the CMV pp65 protein; Flu/M: overlapping peptides from the Influenza matrix protein; CoV-2/S: overlapping peptides from the SARS-CoV-2 spike protein. HIV I+II: mix of ILKEPVHGV HLA-A*0201 epitope from the HIV reverse transcriptase (RT) protein and YVDRFYKTLRAEQASQEV HLA-class II epitope from HIV group-specific antigen (Gag) protein.

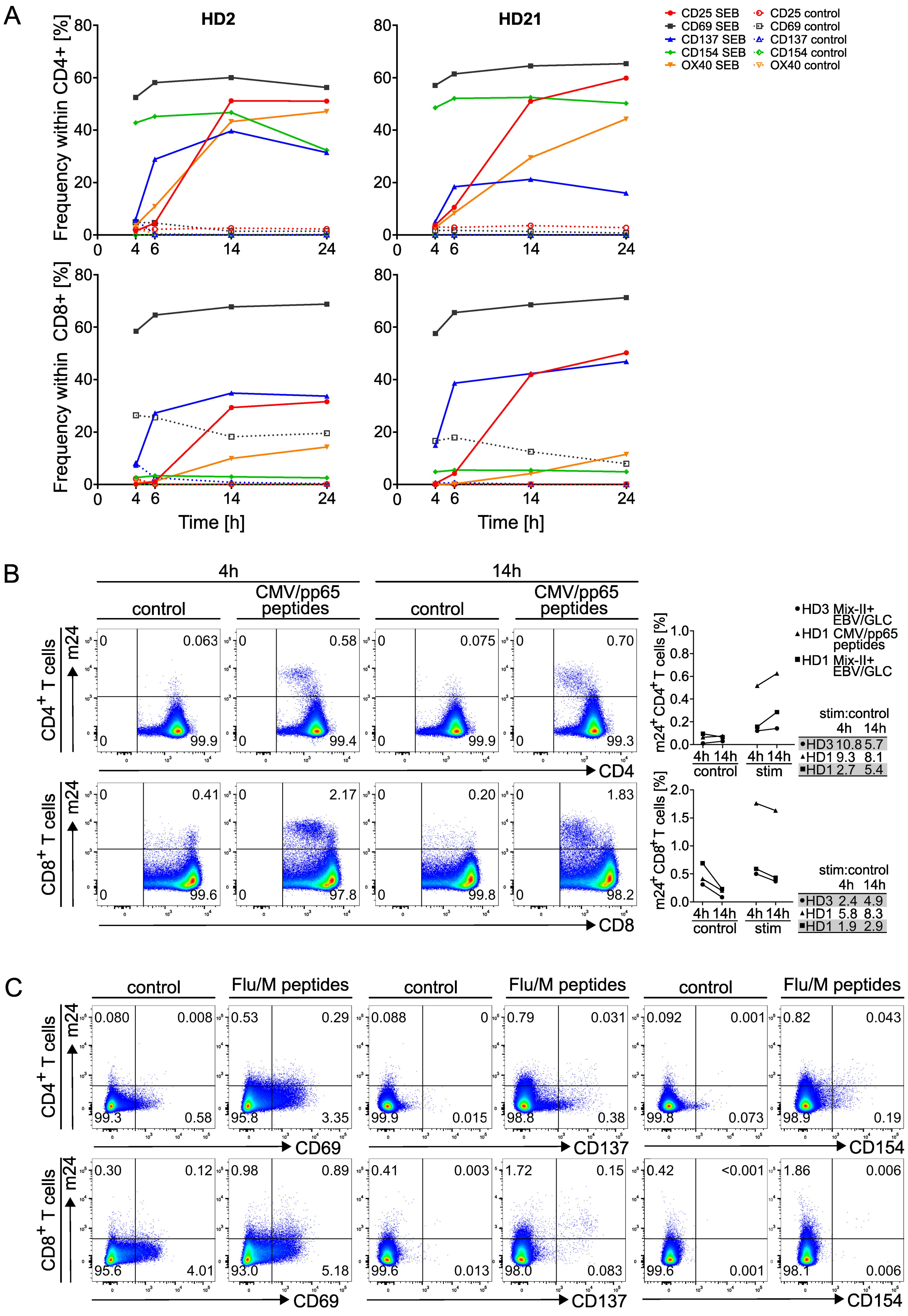
A
HD2
HD21
Frequency within CD4+ [%]
Frequency within CD8+ [%]
Time [h]
CD25 SEB
CD69 SEB
CD137 SEB
CD154 SEB
OX40 SEB
CD25 control
CD69 control
CD137 control
CD154 control
OX40 control
B
4h
14h
control
CMV/pp65 peptides
CD4+ T cells m24
CD8+ T cells m24
CD4
CD8
m24+CD4+ T cells [%]
m24+CD8+ T cells [%]
HD3 Mix-II+ EBV/GLC
HD1 CMV/pp65 peptides
HD1 Mix-II+ EBV/GLC
stim:control
4h 14h
HD3 10.8 5.7
HD1 9.3 8.1
HD1 2.7 5.4
HD3 2.4 4.9
HD1 5.8 8.3
HD1 1.9 2.9
control
stim
C
control
Flu/M peptides
CD69
CD137
CD154

D

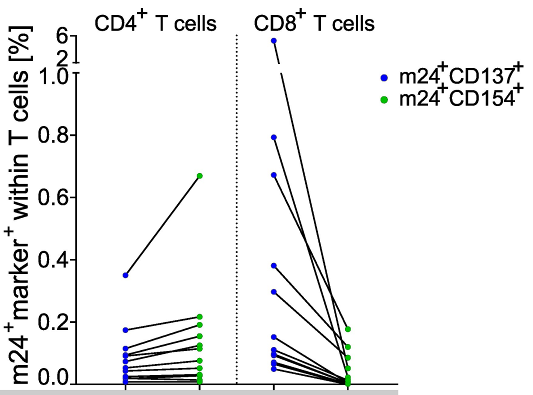

n	12	12	12	12
median	0.063	0.095	0.132	0.009
mean	0.089	0.140	0.673	0.040

Supplementary Figure 1. Activated ß$_2$-integrins are detectable after 14 h of stimulation allowing co-staining with cell surface activation induced markers (AIMs) CD137 and CD154. (A) Time course of CD25 (red), CD69 (black), CD137 (blue), CD154 (green) and OX40 (orange) expression on CD4$^+$ (top) and CD8$^+$ (bottom) T cells after 4, 6, 14 and 24 h of incubation with SEB (solid lines) and on unstimulated PBMCs (dotted line) for two donors (HD2 and HD21). (B) Detection of activated ß$_2$-integrins with m24 Ab staining after 4 vs 14 h of peptide stimulation on CD4$^+$ (top) and CD8$^+$ (bottom) T cells. Exemplary dot plots of donor HD1 after incubation with control or CMV/pp65 overlapping peptides for 4 h (left) vs 14 h (middle). Frequencies of marker$^+$ cells and stimulated:control (stim:control) ratios for n=3 donor/antigen combinations are shown (right). (C) Dot plots of a representative donor (HD4) for combination of m24 Ab staining with detection of CD69 (left), CD137 (middle) and CD154 (right) for CD4$^+$ (top) and CD8$^+$ (bottom) T cells after 14 h of incubation with control or Flu/M overlapping peptides. (D) Comparison of m24$^+$CD137$^+$ and m24$^+$CD154$^+$ detection on CD4$^+$ (left) and CD8$^+$ (right) T cells after 14 h of peptide stimulation with mean and median of detected frequencies. n=12 donor/antigen combinations are included. Frequencies of stimulated samples are background subtracted (A, B and D).

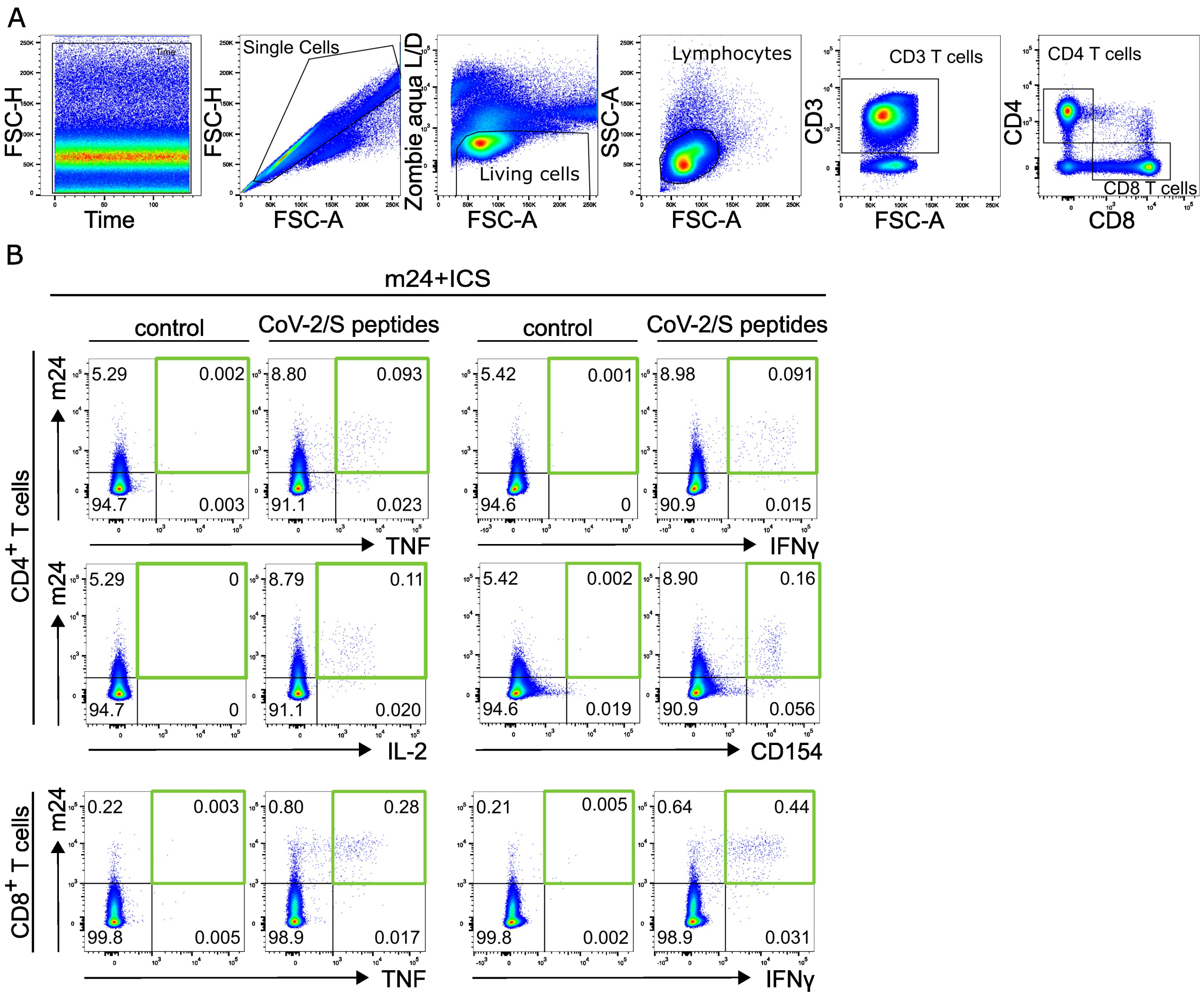

Supplementary Figure 2. (A) Main gating strategy. From left to right: time gate, FSC-A/FSC-H duplet exclusion (single cell gate), Zombie aqua/ FSC-A dead cell exclusion (living cell gate), SSC-A/FSC-A lymphocytes gate, CD3^{+} T cells, followed by gating for CD4^{+} and CD8^{+} T cells. (B) Combined gating of m24 Ab staining with intracellular TNF, IFNγ, IL-2 and CD154 expression (m24+ICS) for CD4^{+} (top), and intracellular TNF and IFNγ expression for CD8^{+} (bottom) T cells of donor HD5 stimulated with SARS-CoV-2/S overlapping peptides or left unstimulated. Numbers represent frequencies within CD4^{+} or CD8^{+} T cells. The green squares mark the populations of interest. The corresponding m24+AIM staining of donor HD5 is shown in Figure 1B.

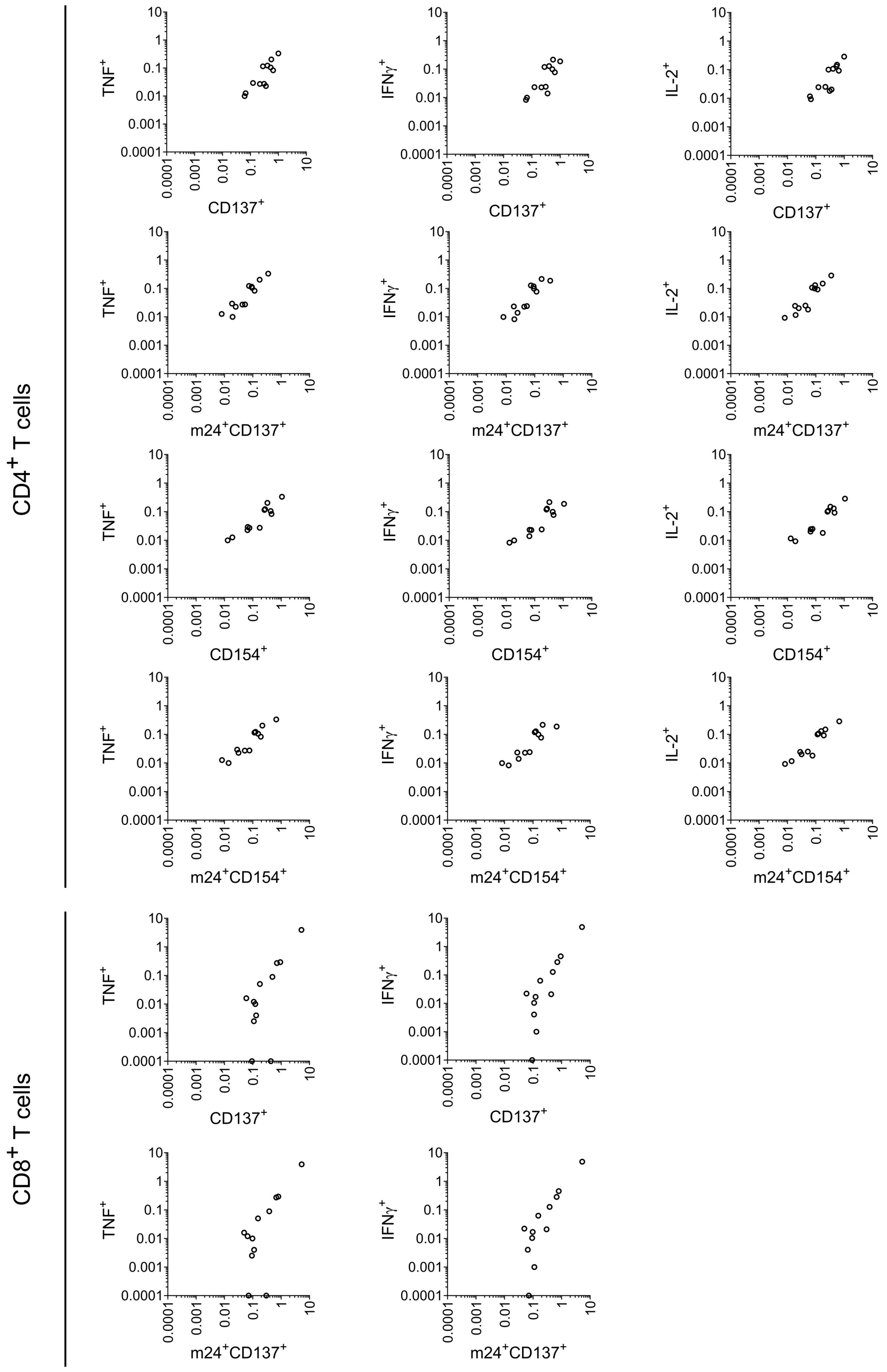

Supplementary Figure 3. Correlations between antigen-specific T cell frequencies assessed by AIM or ICS. Frequencies of antigen-specific CD4^{+} (top) and CD8^{+} (bottom) T cells detected by single AIMs or m24 Ab staining in combination with one AIM were compared to single cytokine expression (n=12 donor/antigen combinations, frequencies of stimulated samples are background subtracted). Values that were 0 were set to 0.0001. Corresponding Spearman r and x-intercepts are presented in Figure 4B.

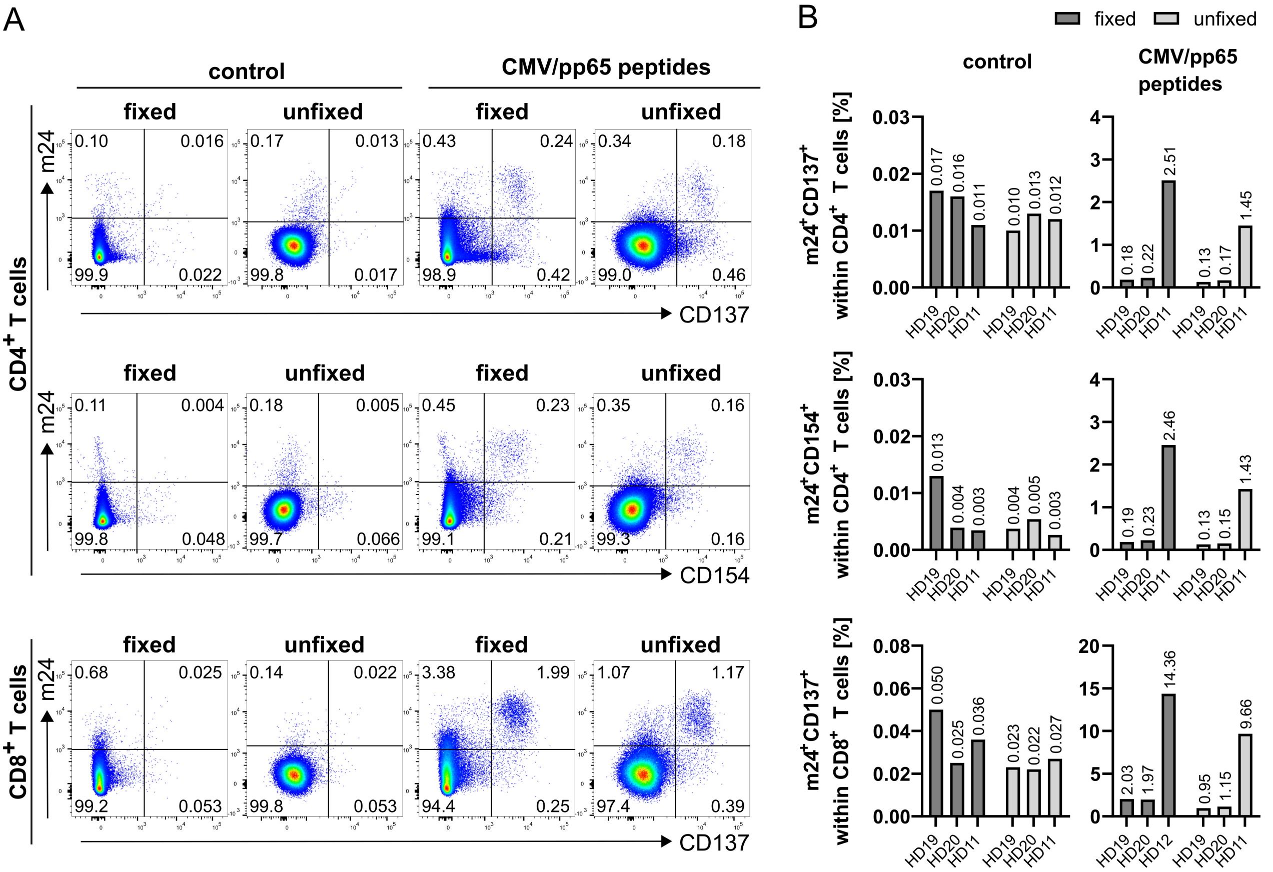

Supplementary Figure 4. Staining of activated β_2-integrins in combination with CD137 or CD154 detection (m24+AIM) is feasible without cell fixation. (A) Dot plots of a representative donor (HD20) for combination of m24 Ab with CD137 and CD154 for CD4$^+$ (top) or m24 Ab with CD137 for CD8$^+$ (bottom) T cells after 14h incubation with control or CMV/pp65 overlapping peptides. Cells were either fixed with FACS-Lysing solution or left unfixed after the staining procedure. Without fixation, cells were centrifuged once after staining, resuspended in FACS buffer and immediately acquired at the flow cytometer. Fixation was performed as described in the section 2.8. (B) Frequencies of m24$^+$CD137$^+$ and m24$^+$CD154$^+$ within CD4$^+$ T cells (top and middle) and m24$^+$CD137$^+$ within CD8$^+$ T cells (bottom) of unstimulated control samples (left) or after stimulation with CMV/pp65 overlapping peptides (right; frequencies of stimulated samples are background subtracted) are plotted for n=3 donors.).
